# Hepatocyte-enriched LEAP2 in relation to heel bone density and osteoporosis-related phenotypes: an exploratory genetic and translational study

**DOI:** 10.3389/fendo.2026.1959499

**Published:** 2026-10-30

**Authors:** Zhicong Xia, Guowen Bai, Xintian Zhang, Yueping Cai, Hongbo Wang, Xiaosheng Lin

**Affiliations:** 1 Shenzhen Clinical College of Integrated Traditional Chinese and Western Medicine, Guangzhou University of Chinese Medicine, Shenzhen, China; 2 Department of Spinal Orthopedics, Shenzhen Hospital of Integrated Traditional Chinese and Western Medicine, Shenzhen, China; 3 Department of Geriatrics, Bao’an Central Hospital of Shenzhen, Shenzhen, China

**Keywords:** hepatocyte, LEAP2, liver–bone axis, Mendelian randomization, osteoporosis, SuSiE colocalization

## Abstract

**Background:**

Unbiased evaluation of liver-derived factors may clarify endocrine links between hepatic metabolism and skeletal fragility; however, hepatic transcript abundance cannot be assumed to represent circulating protein or direct bone-cell signaling.

**Methods:**

We screened 168 liver-secreted genes and 202 independent liver *cis*-eQTL signals across seven skeletal outcomes using outcome-wise false-discovery-rate control, Steiger direction checks, bilateral sum of single effects (SuSiE) fine-mapping, and a three-edge analysis of liver expression, circulating protein, and heel estimated bone mineral density (eBMD). LEAP2 was then examined in human liver cell-resolved, spatial and bulk datasets, public bone transcriptomes, an exploratory hospital serum cohort [30 osteoporosis (OP) and 30 nonosteoporosis (NOP) participants], and a 2 × 2 high-fat diet (HFD) × orchidectomy (Orx) mouse experiment.

**Results:**

The confirmatory genetic screen was null. In the separately defined exploratory layer, LEAP2–heel eBMD was the sole pair meeting the prespecified bilateral SuSiE criteria (PP.H4 = 0.915327); heel eBMD is quantitative-ultrasound-derived rather than a dual-energy X-ray absorptiometry (DXA) outcome. The MR association remained significant after a *post hoc* Bonferroni correction across all 1,414 signal-outcome combinations (adjusted *p* = 1.39 × 10^−14^). Colocalization was prior-sensitive: PP.H4 was 0.8439 at *p*_12_ = 5 × 10^−6^ and 0.1903 under a deliberately conservative *p*_12_ = 1 × 10^−5^/46 stress test, so no formal family-wise colocalization claim was made. The liver eQTL–circulating pQTL edge was analytically indeterminate because the pQTL model did not converge, and the complete genetic triangle was not supported. Hepatocytes dominated *LEAP2* signal in all three cell-resolved datasets. Serum LEAP2 was lower in OP than NOP participants (median [IQR], 5.336 [3.780–6.083] vs. 8.153 [7.015–9.458] ng/mL; *U* = 854, *p* = 2.33 × 10^−9^), but age imbalance and unavailable metabolic covariates preclude an independent association claim. In mice, HFD and Orx were associated with adverse trabecular phenotypes and lower hepatic *Leap2* and circulating LEAP2.

**Conclusion:**

A null confirmatory screen and prior-sensitive exploratory colocalization nominate LEAP2 for further study but do not establish circulating mediation, direct bone-cell GHSR signaling, diagnostic utility, or a causal effect on bone.

## 1 Introduction

Osteoporosis and fragility fractures arise from interacting skeletal, endocrine, metabolic, and inflammatory pathways. Contemporary clinical guidance emphasizes that bone density is one component of fracture risk and that secondary endocrine and metabolic disorders should be considered (1–6). The liver is a major secretory and metabolic organ, and recent experimental and clinical literature supports bidirectional liver–bone communication and highlights the roles of hepatokines, nutrient handling, inflammation, and chronic liver disease (1–3). However, selecting a plausible hepatokine after observing disease-associated expression can create substantial target-selection bias.

Genetic prioritization can reduce target-selection bias when exposure definitions and inferential boundaries remain explicit. A liver expression quantitative trait locus (eQTL) estimates the consequence of genetically predicted hepatic transcript abundance; it does not by itself prove increased protein secretion, circulating abundance, tissue specificity, or direct action on bone. Mendelian-randomization reporting and regional colocalization therefore require transparent harmonization, direction checks, complete variant-region analysis, and explicit treatment of multisignal loci (7–14).

Liver-expressed antimicrobial peptide 2 (LEAP2) is a cysteine-rich circulating peptide originally isolated from human blood and expressed predominantly in the liver (15). It is an endogenous antagonist and inverse agonist of the growth hormone secretagogue receptor (GHSR), and recent human and experimental studies link LEAP2 to feeding, glycemic regulation, adiposity, insulin sensitivity, and liver metabolic state (16–22). These observations provide metabolic context but do not establish a skeletal role or prove that bone cells express a functional LEAP2 receptor.

We therefore performed a prespecified two-tier screen of liver-secreted genes across seven skeletal outcomes. We then used bilateral sum of single effects (SuSiE) colocalization, a circulating-protein quantitative trait locus (pQTL) triangle, and independent single-cell, single-nucleus, spatial, and bulk-liver transcriptomic datasets to define both the evidence supporting LEAP2 and the boundaries that remain unresolved. Finally, we added exploratory human serum validation and a four-group animal experiment involving high-fat diet (HFD)/orchidectomy (Orx) to test whether circulating or hepatic LEAP2 changes accompany osteoporosis-related skeletal deterioration without presuming a direct bone-cell receptor mechanism.

## 2 Materials and methods

### 2.1 Study design and prespecified evidence hierarchy

The analysis followed an evidence hierarchy defined before each corresponding analysis: candidate-universe construction, liver *cis*-eQTL-bone-outcome Mendelian randomization (MR) screening, bilateral SuSiE colocalization, circulating pQTL triangulation, liver cell-source localization, liver bulk validation, and bone-receptor evaluation. The confirmatory analysis and the subsequent Round 1b exploratory analysis were governed by separate dated protocols retained with the run records. As these materials have not yet been assigned a public persistent identifier, the manuscript does not claim external preregistration. The confirmatory null result was retained and was not overwritten by the exploratory layer. Ordinary single-signal coloc.abf results were retained for audit but could not substitute for bilateral SuSiE. Public transcriptomic analyses were planned before serum results were examined. No imputed expression was used to establish *LEAP2* or GHSR detection, and cells or spatial spots were not treated as independent patients. The resources are summarized in **Table 1** and interpretation boundaries in **Table 2**.

**Table 1:** Datasets and validation cohorts used in the study.

Resource	Modality/comparison	Biological units	Primary role
Broadaway (2024)	Liver *cis*-eQTL meta-analysis	Maximum *n* = 1,183; 202 candidate-set signals	Genetic exposure
Seven prespecified bone GWAS	Osteoporosis, fracture and BMD outcomes (25–28; **Supplementary Table S1**)	Outcome-specific samples; 32,961–438,872	MR and colocalization
deCODE LEAP2	Regional plasma pQTL, aptamer 5708_1	35,559 participants (13–14)	Triangle edges B and C
GSE174748	Human liver snRNA-seq; uninjured vs. NAFLD	11,798 singlets; four donors	Source localization; descriptive disease contrast
GSE330266	Human liver sc/snRNA-seq; control vs. MASLD	81,793 retained cells/nuclei; 10 donors	Independent source replication
GSE192740	Human liver cell/nucleus object	167,598 total; 15,063 whole-liver nuclei from five patients	Independent source replication
GSE192741	Human liver Visium	4,333 spots; five sections; four donors	Tissue detection and zonation audit
GSE207310	Human liver bulk RNA-seq	30 donors	NAFL/NASH validation
GSE126848	Human liver bulk RNA-seq	57 donors	Obesity-aware replication
GSE169396/GSE147287	Human bone/bone-marrow scRNA-seq	51,489 input cells; six donors	GHSR detection boundary
GSE156508	Primary osteoblast microarray	Six fractures; six severe-OA controls	Final GHSR bulk audit
Clinical serum cohort	Hospital-based OP versus NOP comparison	30 OP and 30 NOP participants	Exploratory circulating LEAP2 validation
Mouse HFD/Orx model	Sham, Orx, HFD + Sham, and HFD + Orx	*n* = 8 animals/group for qPCR, serum LEAP2, and micro-CT	Exploratory bone phenotype and hepatic/circulating LEAP2 evaluation

**Table 2:** Evidence statements and permitted interpretation.

Evidence component	Observed result	Permitted statement	Prohibited overinterpretation
Liver eQTL–heel eBMD	PP.H4 = 0.9153 at *p*_12_ = 1 × 10^−5^; PP.H4 = 0.1903 at *p*_12_ = 1 × 10^−5^/46	High posterior support under the prespecified prior; positive direction, but prior-sensitive under the conservative stress test	Experiment-wide multiplicity-robust or confirmatory causal candidate
Liver eQTL–circulating pQTL	pQTL SuSiE nonconverged; returned posterior values retained for audit only	Protocol pass criteria not met; biological edge indeterminate	Evidence for or against liver-expression–circulating-protein colocalization
Circulating pQTL–heel eBMD	PP.H4 = 0.6667	Moderate/suggestive only	Strong triangle or mediation
Three liver cell-resolved datasets	Hepatocytes dominate *LEAP2* signal	Hepatocyte-enriched	Liver-specific
Human liver Visium	*LEAP2* detected in all donors; discordant zonation	Tissue-level detection; zonation indeterminate	Portal, central-vein or lipid-droplet localization
Two liver bulk cohorts	NAFL higher than relevant controls	Replicated fatty-liver-associated hepatic increase	LEAP2 causes fatty liver or circulating LEAP2 rises
Bone GHSR audit	Zero single-cell counts; bulk near background	No convincing public evidence for direct bone-cell GHSR	Direct LEAP2–GHSR bone mechanism
Human serum LEAP2	Lower LEAP2 in the author-verified 30 OP/30 NOP dataset; pooled T-score correlation but null within groups	Exploratory group-level clinical association	Independent continuous dose–response or diagnostic biomarker
Mouse bone phenotype	HFD + Orx had the lowest BV/TV and highest Tb.Sp; supported interactions were limited to TV and Tb.Sp	Cross-organ phenotype coherence	LEAP2 caused or rescued bone loss
Mouse hepatic LEAP2	Lower hepatic mRNA and serum LEAP2 with HFD/Orx average effects; no supported qPCR interaction	Context-dependent animal association	Universal direction across fatty-liver states

### 2.2 Liver-secreted candidate universe and genetic sources

The defined universe comprised 168 genes: 167 genes meeting liver-source, secretion, circulating-detection, and human-mouse orthology criteria, plus SOST as a positive-control override. Candidate construction used Human Protein Atlas secretome annotations and UniProt protein-localization evidence (23, 24). The universe, inclusion rules, and override were recorded before outcome screening.

Liver *cis*-eQTL instruments were obtained from the Broadaway et al. meta-analysis of four European-ancestry liver cohorts (maximum *n* = 1,183; GRCh37), using reported conditionally independent cis signals (7). Within the defined universe, 202 independent signals were evaluated against seven skeletal outcomes drawn from FinnGen, GEFOS, and GWAS Catalog datasets (25–29; **Supplementary Table S1**).

### 2.3 MR screening, harmonization, and direction checks

Variants and outcome data were harmonized to GRCh37 and to a common effect allele. Ambiguous multiallelic variants, unresolved palindromic variants, and variants with unreliable frequency alignment were excluded. A Wald ratio was used for single independent instruments; fixed-effect inverse-variance weighting was available for multiple interpretable instruments from the same gene. Instrument strength was summarized by the *F* statistic. Steiger tests were required to support the direction from liver expression to skeletal outcome (30). As femoral-neck and lumbar-spine bone mineral density (BMD) files did not provide original beta and standard error, signed statistics were reconstructed from *p*-value, direction, allele frequency, and sample size and were flagged accordingly.

Bone-adverse directions were defined as positive log odds for osteoporosis or fracture and negative beta for BMD traits. Benjamini–Hochberg false discovery rate (FDR) was calculated separately within each outcome with all 202 signals retained in the denominator; untested or nonevaluable signals contributed *p* = 1 (31). The confirmatory screen required a strong *cis*-eQTL, concordant evidence at FDR < 0.05 in at least two outcomes, including one primary outcome, the correct Steiger direction, and strong final SuSiE support. As a *post hoc* global sensitivity analysis for the selected *LEAP2*–heel eBMD association, the MR *p*-value for this association was also Bonferroni-corrected across all 202 × 7 = 1,414 possible signal-outcome tests.

Round 1b was a separately locked exploratory layer. Instruments were required to have *F* > 10 and the correct Steiger direction; they were included if they met at least one of the following rules: any outcome FDR < 0.10 (E1); at least two *p* < 0.05 associations with concordant bone-adverse direction (E2); or one *p* < 0.01 plus another *p* < 0.10 association with concordant direction (E3). These criteria yielded 26 signals before the translational gate and 25 formal genes. Exploratory MR entry alone was not considered evidence of shared causal variation.

### 2.4 Regional fine-mapping and multisignal colocalization

For every MR-supported candidate-outcome pair, all available variants within the gene boundary ± 500 kb were extracted without *p*-value filtering or LD clumping. Liver eQTL and outcome statistics were aligned to the variant order and reference alleles in the 1,000 Genomes Phase 3 European LD (503 individuals) (32). Each pair required at least 500 shared variants. The two traits were fitted separately using susieR::susie_rss, with an iteration ladder of 200, 1,000, 5,000, and 20,000 iterations. Fixed colocalization priors were *p*_1_ = 1 × 10^−4^, *p*_2_ = 1 × 10^−4^, and *p*_12_ = 1 × 10^−5^. Prior sensitivity was evaluated after fitting, without rerunning susie_rss, because the fitted log Bayes factors were retained (11, 12).

A Round 1b pair passed only if both SuSiE models converged, the minimum absolute within-set correlation of the corresponding credible sets was ≥ 0.50, PP.H4 ≥ 0.50, PP.H4 > PP.H3, PP.H4/(PP.H3 + PP.H4) > 0.50, and the result did not depend on an unresolved variant. For the subsequent pQTL triangle, a stronger threshold was defined: PP.H4 ≥ 0.80 and PP.H4/(PP.H3 + PP.H4) > 0.80, with all other requirements unchanged. The E1–E3 entry rules were candidate-enrichment criteria rather than a single procedure calibrated to control a common experiment-wise error rate across seven outcomes. Outcome-wise BH-FDR across all 202 signals therefore applied only to the MR screening stage. As SuSiE colocalization produces Bayesian posterior probabilities rather than frequentist *p*-values, PP.H4 values were not subjected to BH or Bonferroni adjustment. We instead performed a *post hoc* prior-sensitivity analysis while holding the fitted SuSiE log Bayes factors and p1 = p2 = 1 × 10^−4^ fixed. The shared-variant prior *p*_12_ was evaluated at 1 × 10^−5^, 5 × 10^−6^, 1 × 10^−6^, and 1 × 10^−5^/46. The final value was a deliberately conservative multiplicity stress test and was not interpreted as a formally calibrated Bayesian family-wise-error correction. Nonconvergence constituted failure of the protocol-defined analytical pass criteria. Posterior probabilities from a nonconverged model were not treated as stable biological evidence, and such an edge was classified as indeterminate rather than as evidence against colocalization. An apparently favorable ordinary approximate Bayes factor (ABF) result could not rescue the multisignal classification.

### 2.5 Three-edge analysis of liver expression, circulating LEAP2, and heel eBMD

Three pairwise edges were evaluated over the *LEAP2* ± 500-kb region: liver *LEAP2* eQTL versus heel eBMD (edge A), liver LEAP2 eQTL versus deCODE circulating LEAP2 pQTL (aptamer 5708_1; edge B), and the circulating pQTL versus heel eBMD (edge C). The pQTL analysis used complete regional summary statistics and retained both independent *cis* signals; no lead-SNP substitution was permitted. These were three pairwise colocalization analyses, not a formal mediation model. A complete strong triangle required all three edges to meet the predefined strong threshold with coherent direction. If either trait model did not converge, that edge failed the analytical pass criteria and was considered biologically indeterminate rather than evidence of noncolocalization.

### 2.6 Human liver single-cell and single-nucleus analyses

GSE174748 contained 11,980 liver nuclei from two uninjured and two nonalcoholic fatty liver disease (NAFLD) donors. After excluding 182 predicted doublets, 11,798 singlets were analyzed using the submitted cell annotations and uniform manifold approximation and projection (UMAP). Raw counts defined *LEAP2*-positive nuclei (count > 0), total *LEAP2* counts, and hepatocyte pseudo-bulk profiles; submitted normalized data were used for visualization. Raw hepatocyte counts were summed by donor and expressed as log2 counts per million (CPM). The contrast used an exact two-sided permutation of donor labels. Single-cell workflows were implemented with auditable Seurat-compatible objects and metadata (33, 34).

GSE330266 comprised ten donor-specific 10× filtered matrices [six controls and four metabolic dysfunction-associated steatotic liver disease (MASLD) donors]. Cells were retained if they had ≥ 200 detected genes, ≥ 500 counts, mitochondrial fraction ≤ 20%, and donor-specific upper-tail gene/count metrics below the 99.5th percentile, with an absolute detected-gene cap of 8,000. Broad compartments were assigned by the mean log-normalized expression of prespecified canonical marker sets. A hepatocyte label additionally required at least two detected hepatocyte markers. Raw counts defined *LEAP2* positivity and donor-level pseudo-bulk; log-normalized values were used for marker scoring and visualization. The MASLD–control contrast used all C(4,10) = 210 donor-label assignments. UMAP downsampling was used only for visualization.

The deposited GSE192740 object contained 167,598 cells/nuclei, 30,173 genes, 38 samples, and 19 patients. Sample–patient mapping and assay compartments were audited before expression analysis. CD45-positive, CD45-negative, dendritic-cell-enriched, monocyte/macrophage-enriched, and whole-liver-nuclei samples were kept separate. The prespecified source-localization compartment was whole-liver nuclei (15,063 nuclei from five patients). Raw counts defined *LEAP2* positivity, and the stored normalized RNA data were used for visualization. Hepatocyte pseudo-bulk required ≥ 20 hepatocytes per donor. As no reliable disease phenotype was present in the deposited single-nucleus object or its official sample table, no healthy-versus-steatotic test was performed.

Fatty-acid metabolism, cholesterol metabolism, insulin response and, where supported, bile-acid metabolism were the only prespecified pathway sets. Associations with *LEAP2* were evaluated at donor-level pseudo-bulk and treated as descriptive. No WGCNA, machine learning, CellChat, expression imputation, or *post hoc* pathway expansion was used.

### 2.7 Spatial transcriptomic analysis

Matched human liver Visium data were obtained from GSE192741, the spatial subseries paired with GSE192740. The analysis included 4,333 spots across five sections from four donors (two healthy and two steatotic; H35 contributed two sections). Raw-count positive-spot fractions, normalized expression, and donor-level pseudo-bulk log2 CPM were calculated. Spots were never treated as biological replicates.

Zonation was evaluated using prespecified periportal markers (CPS1, ASS1, and HAL) and pericentral markers (GLUL, CYP2E1, and CYP1A2). Within each section, marker expression was standardized, and the zonation axis was defined as the pericentral score minus the periportal score. The bottom and top thirds were labelled portal-like and central-like transcriptomic states, respectively; these labels were not treated as direct histologic identification of portal tracts or central veins. A directional claim required at least three of four donors to agree in both central-minus-portal pseudo-bulk expression and the sign of the within-section *LEAP2*–zonation correlation. Apparent white spaces in histology were not classified as lipid droplets because no validated segmentation was available.

### 2.8 Bulk-liver validation

GSE207310 comprised 30 human liver biopsies (No-nonalcoholic fatty liver (NAFL), *n* = 5; NAFL, *n* = 15; nonalcoholic steatohepatitis (NASH), *n* = 10). Official raw gene counts were normalized using a positive-count median-of-ratios size factor following established RNA-seq principles (35). *LEAP2* (ENSG00000164406) was analyzed as donor-level log2(normalized count + 0.5). Primary analyses were a three-group Kruskal–Wallis test, two-sided Mann–Whitney pairwise contrasts with BH adjustment, and HC3-robust linear models including disease group, tissue preservation, and sex. BMI and age were not available from GEO and could not be adjusted.

GSE126848 contained 57 liver biopsies (normal-weight, *n* = 14; histologically normal obese, *n* = 12; NAFL, *n* = 15; NASH, *n* = 16). Counts were processed identically. Prespecified analyses included a four-group Kruskal–Wallis test, BH-adjusted contrasts of obese versus normal-weight, NAFL versus obese, NASH versus obese, and NASH versus NAFL, and an HC3-robust model with group and sex. The histologically normal obese group was the key control for distinguishing fatty-liver disease from obesity alone.

For cross-cohort display, each NAFL contrast was shown on its original log2 scale with a Welch 95% confidence interval. Effects were not pooled because the cohorts and control definitions differed. PCA and prespecified pathway panels were retained as descriptive dataset context and did not replace the *LEAP2*-focused primary analysis.

### 2.9 Exploratory evaluation of bone-cell GHSR

To define a mechanistic boundary, raw GHSR counts were audited in two independent human bone/bone-marrow single-cell resources: GSE169396 (four femoral-head donors) and GSE147287 (two CD271-positive bone-marrow donors). Detection required raw counts and cross-donor replication; no imputation was allowed. The final prespecified bulk support dataset was GSE156508 (six osteoporotic-fracture and six severe-osteoarthritis primary osteoblast samples on GPL16686). GHSR mapping, absolute signal relative to the platform background, and the group contrast were assessed in that order.

### 2.10 Human serum cohort and LEAP2 measurement

Participants were recruited by hospital-based convenience sampling from orthopedic clinical services at Shenzhen Hospital of Integrated Traditional Chinese and Western Medicine, Shenzhen, China. The final author-verified analysis file included 60 participants: 30 with osteoporosis (OP) and 30 without osteoporosis (NOP). OP classification was supported by the documented clinical diagnosis together with the lowest recorded dual-energy X-ray absorptiometry (DXA) T-score ≤ − 2.5 and/or a clinician-recorded low-trauma fragility fracture. NOP participants did not meet the final OP definition and had no verified fragility fracture. Based on the recorded lowest T-score, the NOP group included 22 participants with normal BMD (T-score ≥ − 1.0) and eight with osteopenia (− 2.5 < T-score < − 1.0). The cohort is presented as an exploratory clinically classified sample rather than a centrally adjudicated diagnostic cohort. Fracture timing and mechanism were not uniformly readjudicated, so fracture frequency was not analyzed as a participant-level outcome. Individuals with other major metabolic bone diseases, documented secondary causes of osteoporosis, active malignancy, severe hepatic or renal dysfunction, acute infection or inflammatory disease, recent exposure to medications with major effects on bone metabolism, incomplete core data, or insufficient serum volume were excluded.

Fasting venous blood was collected in the morning after an overnight fast, processed, aliquoted, and stored until assay. Human serum LEAP2 was measured using the Human LEAP2 ELISA kit. Samples were assayed in three technical wells at a 1:5 dilution and quantified using a four-parameter logistic curve. The manufacturer reported a detection range of 0.312–20 ng/mL, analytical sensitivity/LOD of < 0.078 ng/mL, intra-assay CV of < 8%, inter-assay CV of < 10%, and no significant cross-reactivity or interference with tested human LEAP2 analogues, while noting that cross-reactivity testing was not exhaustive. The lowest calibrator (0.312 ng/mL) was used as the operational lower in-well reportable limit, corresponding to 1.56 ng/mL in original serum after the 1:5 dilution factor. Participant-level means ranged from 1.926 to 11.003 ng/mL after dilution correction; no participant mean fell below this operational limit, and no substitution was required. The participant-level mean was the biological observation. Reporting follows STROBE principles (36). The protocol was approved by the Ethics Committee of Shenzhen Hospital of Integrated Traditional Chinese and Western Medicine (Approval No. KY-2026-031-02; 16 April 2026).

### 2.11 Animal model and experimental groups

Male C57BL/6J mice aged 6–8 weeks were obtained from Guangdong Zhiyuan Laboratory Animal Technology Co. Ltd. (Guangdong, China). Mice were housed under specific-pathogen-free (SPF) conditions with controlled environmental parameters (temperature 22 °C ± 2 °C, relative humidity 50%–60%, and a 12 h light/dark cycle) and had free access to food and water. After a 1-week acclimatization period, mice were randomly assigned to four experimental groups using a computer-generated randomization sequence: Sham, Orx, high-fat diet-fed Sham (HFD + Sham), and high-fat diet-fed Orx (HFD + Orx) groups (*n* = 8 per group). The experimental design followed a 2 × 2 factorial structure, with dietary intervention (standard chow diet or high-fat diet) and surgical intervention (Sham operation or bilateral orchidectomy) as independent factors. The experimental unit was the individual mouse. Mice were housed in individually ventilated cages with four mice per cage, and cage allocation was balanced among groups. Mice in the HFD groups were fed a diet providing 60% of calories from fat (Beijing HFK Bioscience Co. Ltd., Beijing, China; catalogue number: 0260319-01), whereas control mice received a standard chow diet. Dietary intervention was continued for 12 weeks. After 8 weeks of HFD feeding, bilateral orchidectomy was performed to establish an androgen-deficiency-induced osteoporosis model. Mice were anesthetized with sodium pentobarbital (50 mg/kg, intraperitoneal injection). Adequate depth of anesthesia was confirmed by the absence of the pedal withdrawal response to toe pinch before surgery. Bilateral testes were then surgically removed. Sham-operated mice underwent identical surgical procedures without testicular removal. Following surgery, penicillin solution (40,000 IU/mL, 0.2 mL per mouse, intraperitoneal injection) was administered to prevent postoperative infection according to the approved animal protocol.

After orchidectomy, mice were maintained on their respective diets for an additional 4 weeks. Before terminal sampling, mice were fasted for 12 h with free access to water. Terminal sampling and tissue collection were performed the following morning during the light phase. Mice were deeply anesthetized with sodium pentobarbital (50 mg/kg, intraperitoneal injection). Adequate depth of anesthesia was confirmed by the absence of the pedal withdrawal response to toe pinch before cardiac puncture. Blood was subsequently collected by cardiac puncture, and the animals were euthanized by exsanguination while maintained under deep anesthesia. Death was confirmed by the cessation of spontaneous respiration and heartbeat before tissue collection. Femurs, tibiae, liver tissues, and serum were collected for analysis. No animals were excluded from the final analysis, and no unexpected deaths occurred. Orchidectomy-associated bone loss was evaluated by micro-computed tomography and bone phenotypic assessment. Investigators responsible for sample collection, micro-CT quantification, molecular experiments, and statistical analyses were blinded to group allocation using coded sample identifiers until data analysis was complete. All animal experiments were approved by the Experimental Animal Ethics Committee of Guangzhou University of Chinese Medicine (Approval No. 20250424010). Study design and reporting followed ARRIVE 2.0 (37).

### 2.12 Micro-CT, histology, and hepatic LEAP2 assays

Trabecular bone was evaluated by microcomputed tomography at the individual-animal level following rodent micro-CT reporting principles (38). The verified export workbook contained BMD (g/cm^3^), total volume (TV; mm^3^), bone volume BV (mm^3^), BV/TV (%), Tb.Th (mm), and Tb.Sp (mm); bone tissues were fixed, decalcified, paraffin-embedded, and stained with hematoxylin and eosin for qualitative evaluation. Liver LEAP2 immunohistochemistry was interpreted qualitatively. Hepatic *Leap2* mRNA was measured in three technical qPCR wells per mouse and displayed as 2^−ΔΔCt^; statistical inference used ΔCt values in accordance with MIQE principles (39). Circulating LEAP2 was measured with a mouse-specific ELISA in three technical wells per mouse, and the three measurements were averaged to obtain one animal-level mean.

### 2.13 Statistical analysis and reproducibility

For the clinical cohort, continuous variables were summarized as median [interquartile range (IQR)] and mean ± SD at the participant level. OP and NOP groups were compared using two-sided Mann–Whitney *U* tests; sex was compared using Fisher’s exact test. Spearman correlations between LEAP2 and minimum T-score were calculated overall and within each group. Separation was handled using Firth bias-reduced logistic regression with profile penalized-likelihood confidence intervals and likelihood-ratio *p*-values (40, 41). Sensitivity analyses included exact-sex nearest-age matching within 5 years and an HC3-robust linear model. Given the limited covariate overlap and the availability of only seven matched pairs, adjusted and matched estimates were considered exploratory and were not interpreted as independent effects. The exact-sex nearest-age matched sensitivity analysis is shown in **Supplementary Figure S2**.

Animal outcomes were analyzed using 2 × 2 factorial linear models with HFD, Orx, and HFD-by-Orx terms coded − 0.5/+ 0.5. All factorial terms used HC3 heteroscedasticity-consistent covariance and finite-sample *F (1*,28) reference tests (42). Interaction-first interpretation was followed: four predefined simple effects were evaluated when an interaction was supported, with Holm adjustment across the four contrasts. Serum LEAP2 additionally underwent a 49,999-replicate restricted-residual Rademacher wild-bootstrap sensitivity analysis (43). BV/TV was designated the principal micro-CT reporting outcome in this revision; because this hierarchy was not sample-size-defining before the experiment, it remains interpretive. The other five micro-CT outcomes were secondary, with Holm correction across the six-outcome family. qPCR inference used ΔCt; figures display 2^–ΔΔCt^. Individual mice, not technical wells or image fields, were biological replicates.

For public-data analyses, all tests were two-sided unless explicitly stated. Multiple comparisons within a dataset were controlled using Benjamini–Hochberg adjustment. Exact label-permutation tests were used when complete donor-level enumeration was feasible. Genetic analyses used summarized-data MR principles (8, 9), susieR fine-mapping (10), coloc.susie (11), and large-scale pQTL resources (13, 14). Liver cell-source and spatial interpretations were benchmarked against published human liver atlases and spatial resources (44–47). Scripts, protocols, input inventories, run metadata, and session information were preserved.

## 3 Results

### 3.1 A prespecified two-tier screen retained the confirmatory null and identified *LEAP2* only in the exploratory layer

The frozen discovery universe contained 168 liver-secreted genes, 202 independent liver *cis*-eQTL signals, and seven skeletal outcomes (**Figure 1A**). The original confirmatory workflow yielded no H1 or H2 candidates. This null result was retained as the primary confirmatory conclusion.

**Figure 1:**
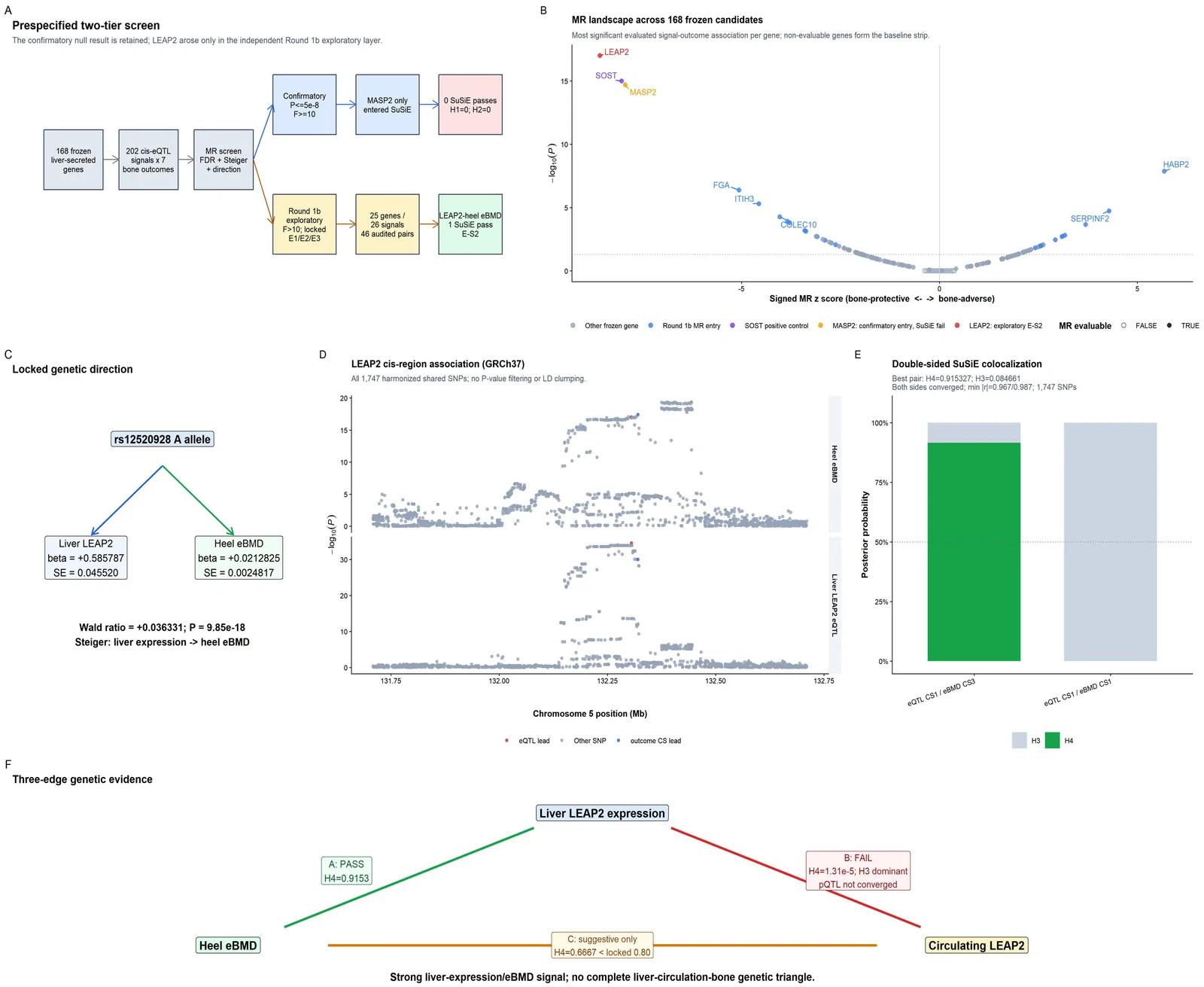
A prespecified two-tier genetic screen retained the confirmatory null and identified *LEAP2* only in the exploratory layer. **(A)** The frozen universe contained 168 liver-secreted genes, 202 independent *cis*-eQTL signals, and seven bone outcomes. The confirmatory analysis retained its null result (H1 = 0; H2 = 0). The separately locked Round 1b exploratory layer generated 46 audited candidate-trait analyses (44 new analyses plus two pre-existing MASP2 analyses), with one SuSiE-positive item: *LEAP2*–heel eBMD. **(B)** MR landscape using the most significant evaluated signal–outcome association for each frozen gene. The *x*-axis is the bone-adverse-oriented MR *Z*-score, which permits display across binary and quantitative outcomes; nonevaluable genes are shown in a baseline strip and do not receive an invented effect estimate. Colors identify prespecified Round 1b entry genes, MASP2 and LEAP2, and the SOST positive control. **(C)** Direction locked before serum measurement. The rs12520928 A allele increased liver *LEAP2* expression and heel eBMD; the Wald ratio was positive, and the Steiger direction was correct. **(D)** Regional association across all 1,747 harmonized shared variants in the *LEAP2 cis* region. No *p*-value filtering or LD clumping was performed. **(E)** Bilateral SuSiE colocalization. The best credible-set pair yielded PP.H4 = 0.915327 and PP.H3 = 0.084661 at the prespecified *p*_12_ = 1 × 10^−5^; both models converged, and the corresponding credible sets exceeded the purity threshold. PP.H4 was 0.8439 at *p*_12_ = 5 × 10^−6^, 0.5195 at *p*_12_ = 1 × 10^−6^, and 0.1903 under the deliberately conservative *p*_12_ = 1 × 10^−5^/46 stress test. **(F)** Three-edge genetic evidence. Liver *LEAP2* expression and heel eBMD colocalized strongly (edge A). The liver eQTL–circulating pQTL edge did not meet the analytical pass criteria because the pQTL SuSiE model did not converge; the H3/H4 values returned are retained for audit only, and the biological edge is indeterminate (edge B). Circulating pQTL–heel eBMD evidence was suggestive but below the predefined strong threshold (edge C). The complete triangle was not supported. *LEAP2* remains Exploratory E-S2 and is not a confirmatory H1/H2 candidate. The complete liver–circulation–bone genetic triangle was not supported. The MR association survived a *post hoc* 1,414-test Bonferroni correction, but no formally calibrated family-wise procedure was applied to the 46 Bayesian colocalization analyses; the 46-fold reduction in the prior probability was a sensitivity stress test rather than a Bayesian Bonferroni correction.

The separately defined Round 1b exploratory criteria admitted 26 signals before the translational gate and 25 formal genes. Forty-four new candidate–outcome regional analyses were completed and combined with two pre-existing MASP2 analyses, yielding 46 audited SuSiE items. All planned new items produced auditable outputs; model nonconvergence was classified as analytical failure rather than replaced by ordinary ABF. No formally calibrated family-wise multiplicity procedure was applied across these 46 exploratory analyses. *LEAP2*–heel eBMD was the sole pair meeting every fixed exploratory SuSiE criterion at the prespecified prior, but this classification was evaluated further by prior-sensitivity analysis and does not establish experiment-wide false-positive control. *LEAP2* was therefore classified as Exploratory E-S2 and was not promoted to confirmatory H1/H2 status (**Figures 1A, B**).

The direction was locked before serum measurement. After harmonization to the rs12520928 A allele, the allele increased liver *LEAP2* expression (*β* = 0.585787, SE = 0.0455199) and heel eBMD (β = 0.0212825, SE = 0.00248172). The Wald ratio was positive (*β* = 0.036331, SE = 0.004237, *p* = 9.85 × 10^−18^); the outcome-wise FDR was 1.99 × 10^−15^; the *post hoc* Bonferroni-adjusted *p*-value across all 1,414 signal–outcome combinations was 1.39 × 10^−14^; and Steiger direction was correct (**Figure 1C**). Thus, genetically predicted higher hepatic *LEAP2* was associated with higher heel eBMD.

### 3.2 Multisignal colocalization supported the liver eQTL–heel eBMD edge but not a complete genetic triangle

Regional analysis retained all 1,747 harmonized variants shared between the *LEAP2* liver eQTL and heel eBMD datasets (**Figure 1D**). The liver eQTL and heel eBMD SuSiE fits converged in six and nine iterations and produced one and two credible sets, respectively. The best credible-set pair yielded PP.H4 = 0.915327, PP.H3 = 0.084661, and PP.H4/(PP.H3 + PP.H4) = 0.915338. Minimum absolute correlations were 0.967 for the liver eQTL credible set and 0.987 for the corresponding heel eBMD credible set. The second eBMD credible-set pairing was H3-dominant, reinforcing the need for signal-pair-specific rather than region-wide interpretation (**Figure 1E**).

Prior-sensitivity analysis retained the same credible-set pair but showed that the posterior classification depended on the shared-variant prior. PP.H4 was 0.9153 at the prespecified *p*_12_ = 1 × 10^−5^, 0.8439 at *p*_12_ = 5 × 10^−6^, and 0.5195 at *p*_12_ = 1 × 10^−6^. Under the deliberately conservative *p*_12_ = 1 × 10^−5^/46 stress test, PP.H4 fell to 0.1903 and no longer met the exploratory threshold. Thus, the MR association was robust to the global Bonferroni sensitivity analysis, whereas the colocalization classification was not robust to the most conservative multiplicity-aware prior specification.

The liver eQTL–circulating pQTL edge did not meet the protocol-defined analytical criteria and was biologically indeterminate. Among 1,520 shared variants, the liver eQTL model converged, whereas the pQTL model completed all 20,000 permitted iterations and returned converged = FALSE. A sufficiently resolved ELBO trace was not retained to determine whether the objective was still improving at termination; we therefore do not infer whether additional iterations would have changed the result. The nonconverged fit returned a maximum multisignal PP.H4 of 1.31 × 10^−5^ and PP.H3 of 0.999987; these posterior values are retained for audit only and are not treated as stable evidence against colocalization. Ordinary ABF gave PP.H4 = 0.928, but a single-signal result could not replace the nonconverged multisignal analysis under the defined protocol. The circulating pQTL–heel eBMD edge included 1,560 shared variants and converged bilaterally; its maximum PP.H4 was 0.666720 with PP.H3 = 0.333240. This was moderate evidence but did not meet the 0.80 threshold for strong evidence. Accordingly, the complete liver-expression–circulating-protein–heel-eBMD genetic triangle was not supported (**Figure 1F**).

### 3.3 Three independent human liver datasets identified hepatocytes as the dominant *LEAP2* source

In GSE174748, 11,798 singlet nuclei from four donors were retained (**Figures 2A–C**). Hepatocytes comprised 7,904 nuclei; 4,314 (54.58%) were *LEAP2*-positive. Hepatocytes accounted for 95.98% of all *LEAP2* raw counts, and the median donor-specific *LEAP2*-positive hepatocyte fraction was 54.17%. The donor-level hepatocyte pseudo-bulk difference for NAFLD versus uninjured liver was + 0.984 log2 CPM (1.98-fold; exact *p* = 0.333). Given that each group contained only two donors, this contrast was descriptive.

**Figure 2:**
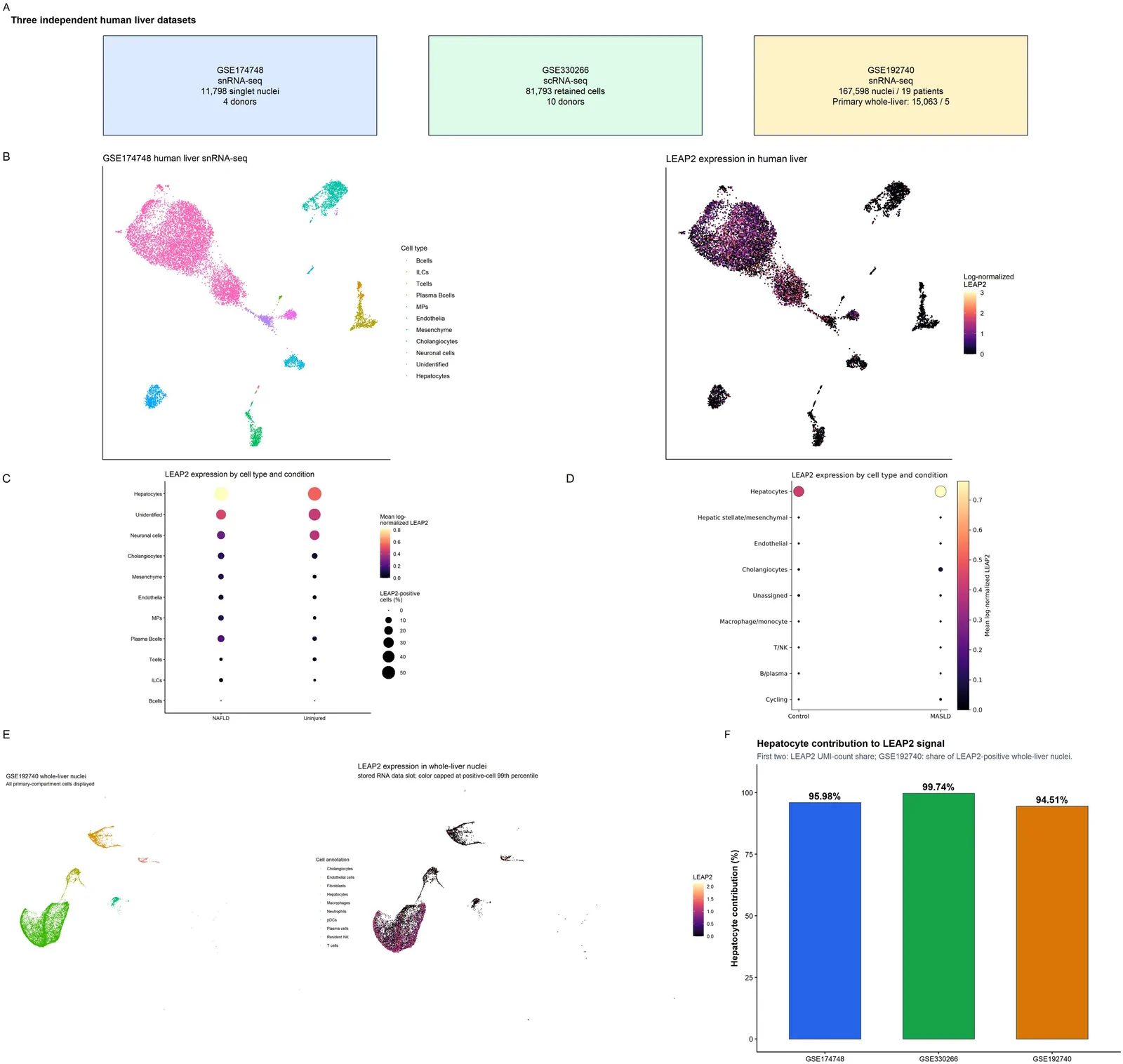
Three independent human liver single-cell or single-nucleus datasets support hepatocyte-enriched *LEAP2* expression. **(A)** Dataset and donor composition. For GSE192740, the full object is reported for audit, whereas the primary source-localization compartment was the whole-liver subset (15,063 nuclei from five patients); CD45-enriched compartments were not pooled into group inference. **(B, C)** GSE174748 cell-type UMAP, *LEAP2* FeaturePlot, and cell-type DotPlot. **(D)** Compact independent replication in GSE330266. **(E)** GSE192740 whole-liver nuclei UMAP and LEAP2 FeaturePlot. Of 10,991 hepatocytes, 3,409 were *LEAP2*-positive (31.02%); hepatocytes comprised 94.51% of *LEAP2*-positive whole-liver nuclei. **(F)** Hepatocyte source metrics across datasets. GSE174748 and GSE330266 report the hepatocyte share of *LEAP2* UMI counts (95.98% and 99.74%); GSE192740 reports the hepatocyte share of *LEAP2*-positive nuclei (94.51%). These related but nonidentical metrics are used only for source localization. Conclusion: *LEAP2* is hepatocyte-enriched; liver specificity is not claimed.

GSE330266 provided an independent 10-donor replication (**Figure 2D**). Of the 93,773 initial barcodes, 81,793 passed the locked quality-control rules; 58,426 were annotated as hepatocytes. Hepatocytes contained 99.74% of all *LEAP2* raw counts, with 27,202 *LEAP2*-positive cells (46.56%) and a median donor-positive fraction of 47.65%. Hepatocyte pseudo-bulk *LEAP2* was + 1.792 log2 CPM in MASLD versus control donors (3.46-fold; exact *p* = 0.0619). This trend did not meet conventional significance and was not used to modify the genetic direction.

The full GSE192740 object contained 167,598 cells/nuclei from 19 patients, but compartment audit identified 15,063 whole-liver nuclei from five patients as the coherent primary source-localization set (**Figures 2A, E**). Of the 10,991 hepatocytes, 3,409 were *LEAP2*-positive (31.02%). Hepatocytes accounted for 94.51% of all *LEAP2*-positive nuclei in this compartment; the next contributions were endothelial cells (2.36%), fibroblasts (1.61%), cholangiocytes (0.86%), and macrophages (0.67%). Disease-group inference was not performed because the single-nucleus metadata lacked a reliable condition label. Collectively, all three datasets supported hepatocyte-enriched—not liver-specific—*LEAP2* expression (**Figure 2F**). The first two percentages in **Figure 2F** represent count shares, whereas the percentage for GSE192740 represents the proportion of positive nuclei; these metrics were not meta-analyzed.

### 3.4 Spatial transcriptomics confirmed tissue-level detection but not a reproducible lobular zone

*LEAP2* was spatially detected in all five Visium sections and all four donors (**Figures 3A, B**). Donor-level *LEAP2*-positive spot fractions ranged from 91.98% to 95.29%, and donor pseudo-bulk abundance ranged from 7.74 to 9.00 log2 CPM. Representative H36 (healthy) and H37 (steatotic) sections were selected for tissue completeness and visualization quality rather than for the magnitude of a disease difference.

**Figure 3:**
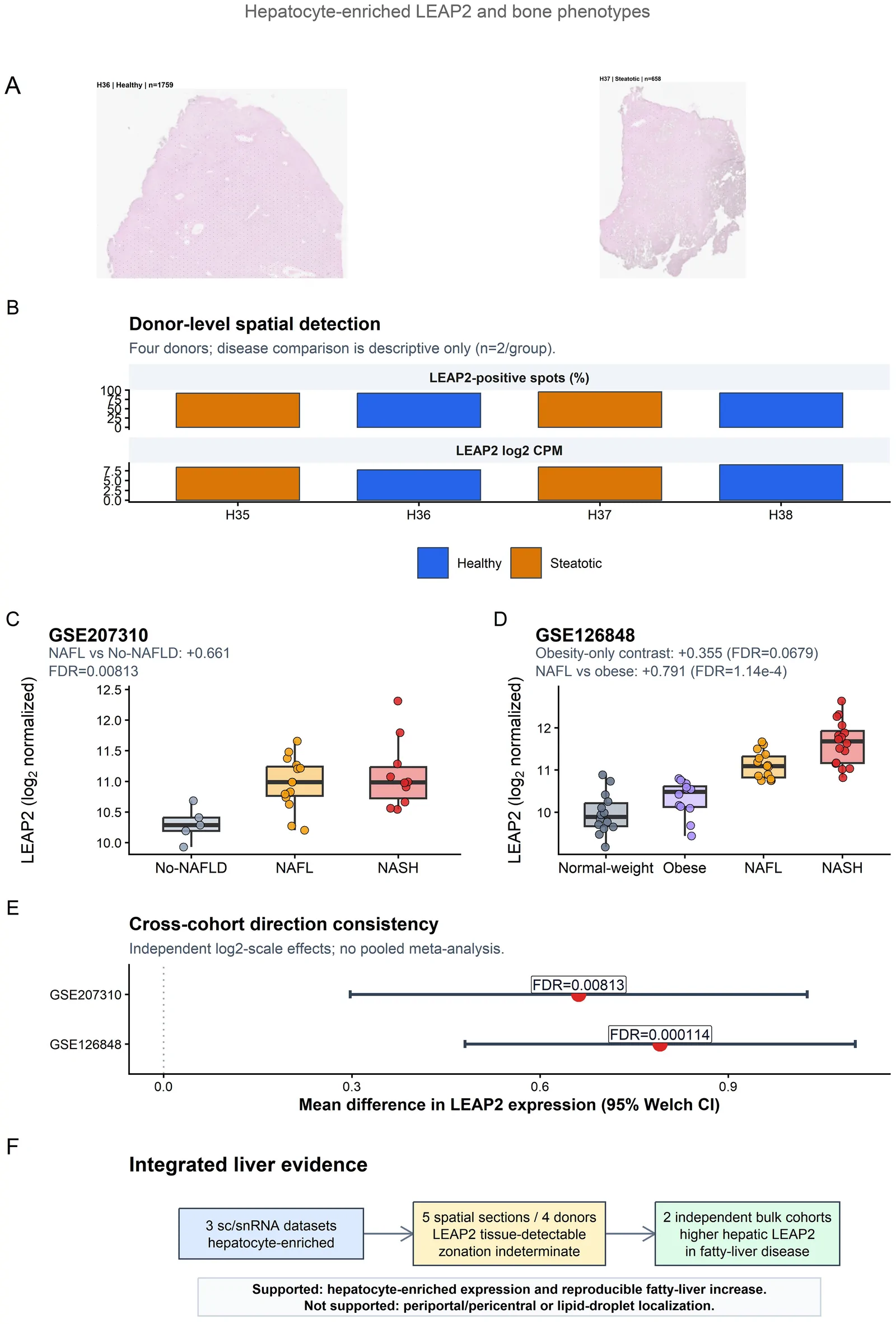
Spatial detection and bulk-liver validation of *LEAP2*. **(A)** Representative complete Visium tissue sections from one healthy (H36) and one steatotic (H37) donor used for spatial transcriptomic analysis. Selection was based on section completeness and visualization quality, not disease effect. **(B)** Donor-level *LEAP2* expression and positive-spot proportions across all four donors. With only two donors per group, no inferential disease comparison is shown. **(C, D) **Donor-level hepatic *LEAP2* expression in GSE207310 and GSE126848. *LEAP2* expression in NAFL samples was higher than the relevant control group in both cohorts. **(E)** Independent log2-scale effects and Welch 95% confidence intervals. Effects were not pooled across cohorts. **(F)** Integrated liver evidence: hepatocyte-enriched expression in sc/snRNA-seq, tissue-level detection by spatial transcriptomics and repeated fatty-liver-associated upregulation in bulk liver. Spatial zonation was formally indeterminate because donor and metric results were discordant. The data do not support periportal, pericentral, or lipid-droplet localization and do not establish a causal fatty-acid-metabolism mechanism. Histology images in panel A were adapted from Guilliams et al. (44), GEO accession GSE192741 (H36, GSM5764426; H37, GSM5764427), under the Creative Commons Attribution 4.0 International license (CC BY 4.0). The source sections were selected and arranged for the present analysis.

All six prespecified zonation markers passed the technical coverage rule. Nevertheless, *LEAP2* direction was discordant: one donor was pericentral-like, two were periportal-like, and one was discordant between metrics. The prespecified consensus criterion was therefore not met, and zonation was classified as indeterminate. The steatotic-minus-healthy donor difference was only + 0.054 log2 CPM and was descriptive because there were two donors per group. No claim was made for portal, central-vein, or lipid-droplet localization.

### 3.5 Two independent bulk-liver cohorts replicated higher hepatic LEAP2 in fatty liver

In GSE207310, hepatic LEAP2 differed across No-NAFLD, NAFL, and NASH groups (Kruskal–Wallis *p* = 0.0133; **Figure 3C**). Relative to No-NAFLD, NAFL was higher by 0.661 log2 units (FDR = 0.00813), and NASH was higher by 0.810 log2 units (FDR = 0.00813). NASH and NAFL did not differ (Δ = 0.149 log2, FDR = 0.760). In the prespecified robust ordinal model adjusted for tissue preservation and sex, LEAP2 increased by 0.360 log2 units per disease stage (HC3 *p* = 0.00397). BMI and age were unavailable and remained unadjusted limitations.

GSE126848 independently separated normal-weight, histologically normal obese, NAFL, and NASH liver (global *p* = 2.83 × 10^−9^; **Figure 3D**). Obesity alone produced a smaller, nonsignificant increase versus normal weight (Δ = 0.355 log2, FDR = 0.0679). By contrast, NAFL was higher than the obese control group by 0.791 log2 units (FDR = 1.14 × 10^−4^), and NASH was higher than the obese control group by 1.320 log2 units (FDR = 3.71 × 10^−5^). NASH also exceeded NAFL by 0.529 log2 units (FDR = 0.00555). Sex-adjusted robust estimates remained directionally consistent.

The NAFL effect was therefore positive in both cohorts despite different control definitions (**Figure 3E**). Given that the expression scales and comparator groups were not identical, effects were displayed independently and were not pooled. Prespecified pathway correlations did not reproduce consistently across cohorts: the insulin-response score was inversely associated with LEAP2 only in GSE126848 (Spearman *ρ* = − 0.672, FDR = 3.20 × 10^−8^), whereas fatty-acid and cholesterol pathways were not supported in either cohort. These panels were treated as contextual, not mechanistic, evidence. The integrated liver evidence is summarized in **Figure 3F**.

### 3.6 Public bone transcriptomes did not support a direct bone-cell GHSR mechanism

GHSR was present in the feature annotations of the two human bone single-cell datasets, but raw GHSR counts were zero in all 51,489 filtered-matrix cells from six independent donors. Thus, no osteogenic, osteocytic, monocyte/macrophage, or osteoclast-lineage compartment met the prespecified cross-dataset donor-level detection rule. In GSE156508, the GHSR transcript cluster mapped unambiguously, but its entire intensity range (1.82–2.47) overlapped the platform-wide low-intensity mode and could not be shown to exceed background. The osteoporotic-fracture versus severe-osteoarthritis contrast was null (mean difference − 0.068; 95% CI, − 0.345 to 0.209; Welch *p* = 0.586) and was retained for audit only. These data did not establish direct bone-cell GHSR expression, and no substitute receptor was sought (**Supplementary Figure S1**).

### 3.7 Serum LEAP2 was lower in osteoporosis, whereas the overall T-score correlation was driven by group separation

The final author-verified serum dataset contained 30 OP and 30 NOP participants; the NOP group comprised 22 participants with recorded normal BMD and eight with osteopenia (**Table 3**). OP participants were older (median [IQR], 66.0 [61.25–75.0] vs. 47.5 [42.0–52.0] years; *U* = 57, *p* = 6.13 × 10^−9^), with 5 men/25 women in OP and 11 men/19 women in NOP. Serum LEAP2 was lower in OP (5.336 [3.780–6.083]) than in NOP (8.153 [7.015–9.458] ng/mL; *U* = 854, *p* = 2.33 × 10^−9^; **Figure 4A**). No participant mean fell below the operational lower reportable limit. The full-cohort LEAP2-minimum-T-score association was positive (Spearman rho = 0.678), but was absent within NOP (rho = − 0.065, *p* = 0.735) and OP (rho = 0.046, *p* = 0.811), indicating group separation (**Figure 4B**). In the age- and sex-adjusted exploratory Firth model, higher LEAP2 was associated with lower odds of OP classification (OR = 0.220 per 1 ng/mL; 95% CI, 0.033–0.540; *p* = 4.27 × 10^−5^), while age was positively associated with OP (OR = 1.202 per year; *p* = 2.06 × 10^−4^) (**Figure 4C**). The association persisted in this exploratory adjusted model; however, limited covariate overlap, marked age imbalance, unavailable BMI and metabolic covariates, and the absence of central age-appropriate DXA readjudication preclude an independent association claim. Exact-sex nearest-age matching yielded seven pairs; the directionally consistent paired comparison is shown in **Supplementary Figure S2**.

**Table 3:** Exploratory clinical cohort characteristics.

Characteristic	NOP (*n* = 30)	OP (*n* = 30)	*p*-value/test
Age (years)	47.5 [42.0−52.0]	66.0 [61.25−75.0]	*U* = 57; *p* = 6.13 × 10^−9^
Male/female	11/19	5/25	Fisher *p* = 0.143
Minimum T-score	0.30 [− 1.15, 0.975]	− 3.45 [− 3.90, − 2.825]	*U* = 900; *p* = 2.78 × 10^−11^
Serum LEAP2 (ng/mL)	8.153 [7.015–9.458]	5.336 [3.780–6.083]	*U* = 854; *p* = 2.33 × 10^−9^

Values are median [interquartile range] unless indicated otherwise. Sex is reported as male/female counts. Continuous variables were compared using two-sided Mann–Whitney U tests; sex was compared using Fisher’s exact test. This note matches the existing Methods and does not change any data.

**Figure 4:**
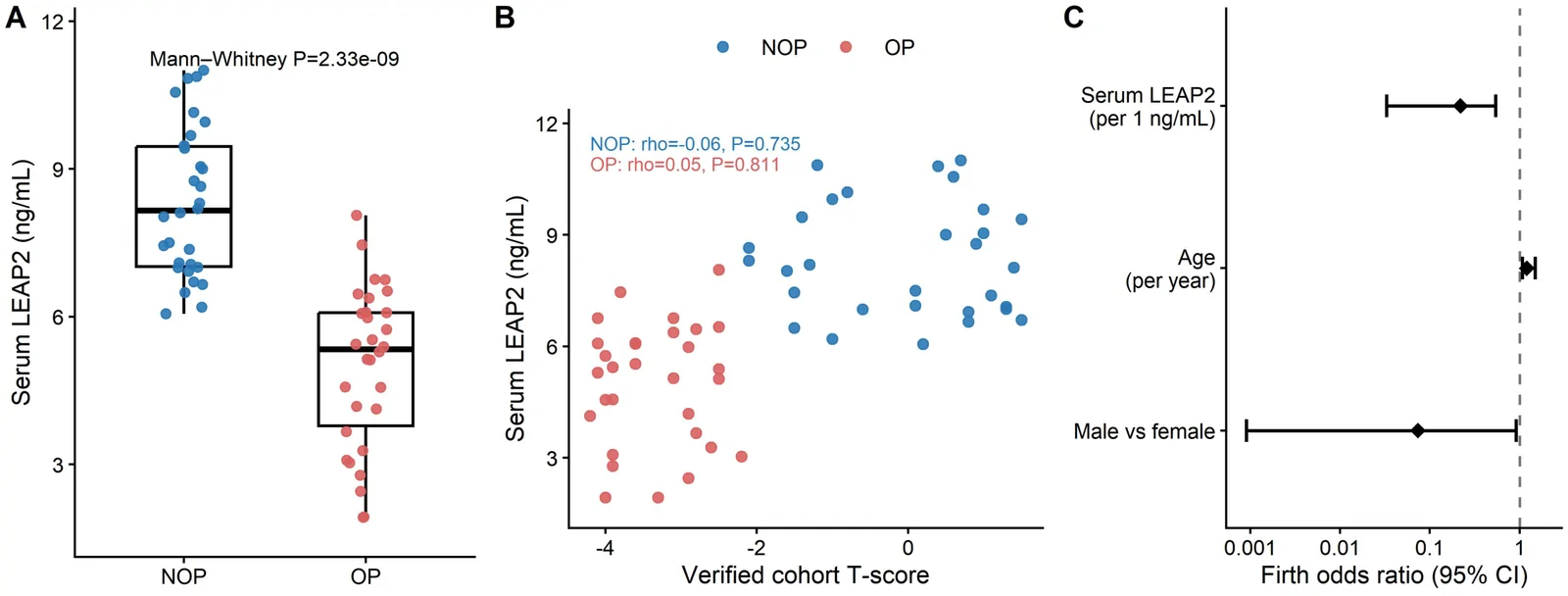
Exploratory clinical association of circulating LEAP2 with osteoporosis status. **(A)** Serum LEAP2 concentrations in NOP (*n* = 30) and OP (*n* = 30). All 60 participant-level means are shown; groups were compared using a two-sided Mann–Whitney *U* test. Concentrations are dilution-corrected and expressed in nanograms per milliliter, and no participant mean fell below the operational lower reportable limit. **(B)** Serum LEAP2 versus minimum T-score, colored by group. No pooled regression line is shown; group-stratified Spearman estimates demonstrate that the overall association was mainly due to between-group separation. **(C)** Exploratory Firth logistic regression adjusted for age and sex, with profile penalized-likelihood 95% confidence intervals. Limited covariate overlap and residual confounding preclude an independent association claim.

### 3.8 HFD and orchidectomy were associated with trabecular bone deterioration

Representative micro-CT reconstructions (**Figure 5A**) showed adverse trabecular patterns in Orx- and HFD-exposed mice, most pronounced in HFD+Orx. HFD+Orx had the lowest BV/TV (9.36 ± 2.17%) and highest Tb.Sp (0.418 ± 0.025 mm). Among six verified micro-CT outcomes (**Figures 5D–I**; **Table 4**), HC3 testing supported HFD-by-Orx interactions for TV (*p* = 5.74 × 10^−7^; Holm *p* = 3.45 × 10^−6^) and Tb.Sp (*p* = 1.41 × 10^−6^; Holm *p* = 7.04 × 10^−6^), but not BMD (*p* = 0.0651), BV (*p* = 0.830), BV/TV (*p* = 0.616), or Tb.Th (*p* = 0.128). For TV, the Orx effect was not supported under chow (Holm *p* = 0.496) but was large under HFD (+ 1.581 mm^3^; Holm *p* < 1 × 10^−10^). For Tb.Sp, both Orx and HFD simple effects were supported in both strata after Holm correction. Bone H&E staining (**Figure 5B**) provided qualitative morphologic context only.

**Figure 5:**
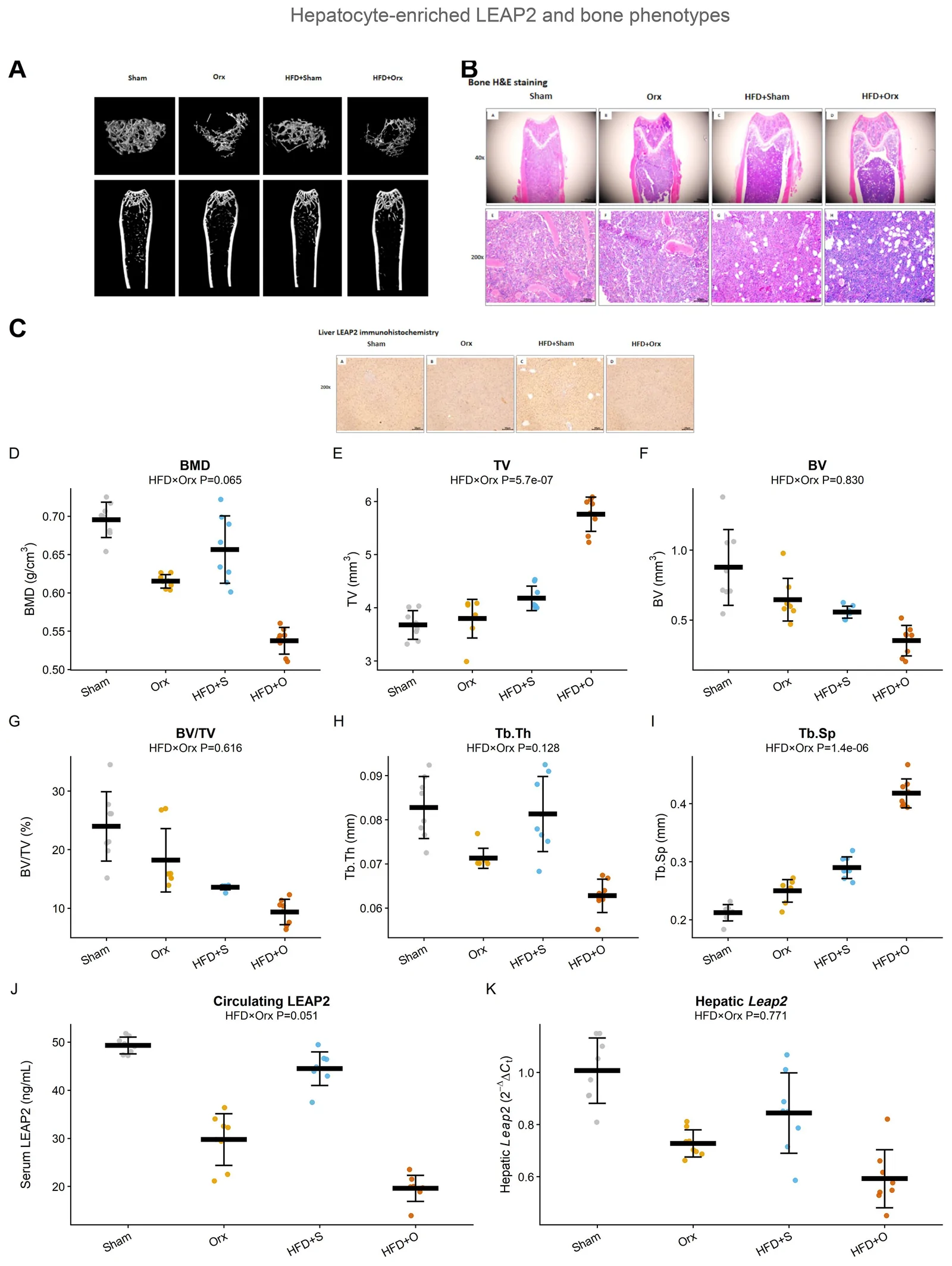
Trabecular phenotype and hepatic/circulating LEAP2 in the HFD × orchidectomy mouse experiment. **(A)** Representative micro-CT 3D reconstructions and longitudinal coronal sections. **(B)** Representative bone H&E staining. **(C)** Representative qualitative liver LEAP2 immunohistochemistry; no animal-level staining inference was performed. **(D–I)** Verified micro-CT outcomes: BMD, TV, BV, BV/TV, Tb.Th, and Tb.Sp. **(J)** Mouse serum LEAP2 (ng/mL). **(K)** Hepatic *Leap2* relative expression. All animal-level points are shown with mean ± SD (*n* = 8/group). **(D–I, K)** HC3 HFD-by-Orx interaction *p*-values; **(J)** the 49,999-replicate wild-bootstrap interaction *p*-value. Interaction-first simple effects and multiplicity handling are described in Methods and **Supplementary Table S2**. No Games–Howell inference is used.

**Table 4:** Exploratory factorial analyses in the mouse experiment.

Outcome	HFD HC3 *p*-value	Orx HC3 *p*-value	HFD × Orx HC3 *p*-value	Multiplicity/sensitivity
Hepatic *Leap2* ΔCt	0.00133	2.04 × 10^−6^	0.771	Inference on ΔCt
Serum LEAP2 (ng/mL)	6.68 × 10^−6^	6.99 × 10^−16^	0.0614	Wild-bootstrap interaction *p* = 0.0508
BMD	3.38 × 10^−6^	1.31 × 10^−10^	0.0651	Holm interaction *p* = 0.261
TV	1.61 × 10^−11^	3.88 × 10^−8^	5.74 × 10^−7^	Holm interaction *p* = 3.45 × 10^−6^
BV	3.96 × 10^−5^	0.00176	0.830	Holm interaction *p* = 1.000
BV/TV	1.29 × 10^−6^	0.00361	0.616	Principal reporting outcome; Holm interaction *p* = 1.000
Tb.Th	0.0346	2.89 × 10^−7^	0.128	Holm interaction *p* = 0.385
Tb.Sp	5.29 × 10^−16^	7.90 × 10^−12^	1.41 × 10^−6^	Holm interaction *p* = 7.04 × 10^−6^

HC3 F tests used *df* = 1, 28. Holm correction was applied across the six micro-CT outcomes for each factorial term. Interaction-first simple effects were prespecified for supported interactions. Serum LEAP2 interaction sensitivity used 49,999 wild-bootstrap replicates.

### 3.9 Hepatic *Leap2* mRNA and circulating LEAP2 were reduced after Orx and HFD exposure

Representative liver immunohistochemistry (**Figure 5C**) suggested qualitative between-group staining patterns but was not used for inferential claims. Hepatic *Leap2* relative expression was 1.007 ± 0.125 in Sham, 0.728 ± 0.052 in Orx, 0.844 ± 0.154 in HFD + Sham, and 0.592 ± 0.111 in HFD + Orx (**Figure 5K**). HC3 testing on ΔCt supported average HFD (*p* = 0.00133) and Orx (*p* = 2.04 × 10^−6^) effects without interaction (*p* = 0.771). Serum LEAP2 was 49.32 ± 1.74, 29.74 ± 5.38, 44.48 ± 3.48, and 19.61 ± 2.72 ng/mL, respectively (**Figure 5J**). HC3 tests supported HFD (*p* = 6.68 × 10^−6^) and Orx (*p* = 6.99 × 10^−16^) effects; the interaction did not reach the 0.05 significance threshold in either the HC3 (*p* = 0.0614) or wild bootstrap (*p* = 0.0508) analysis. Given that factorial testing supported independent Orx effects on hepatic *Leap2* mRNA and circulating LEAP2, the mouse reduction cannot be attributed to HFD or steatosis alone. These findings differ from higher hepatic *LEAP2* in two human fatty-liver bulk cohorts and do not represent a universal fatty-liver direction.

### 3.10 Pooled liver–serum–bone correlations were mainly attributable to between-group separation

Across all 32 mice, hepatic *Leap2* relative expression correlated with serum LEAP2 (Spearman *ρ* = 0.723, FDR-adjusted *p* = 8.25 × 10^−6^). Serum LEAP2 correlated positively with BV/TV (*ρ* = 0.879, FDR *p* = 3.14 × 10^−10^) and negatively with Tb.Sp (*ρ* = − 0.786, FDR *p* = 4.18 × 10^−7^), whereas the serum LEAP2–BMD correlation was not significant (*ρ* = 0.320, *p* = 0.0745; **Supplementary Figure S3**). However, within-group correlations were inconsistent, and the coefficients for serum LEAP2 or hepatic *Leap2* mRNA were attenuated in models adjusted for HFD, Orx, and their interaction. These analyses therefore support cross-layer covariation across experimental conditions, not independent individual-level coupling or mediation.

## 4 Discussion

### 4.1 Principal findings

This study used a two-tier design to move from a defined liver-secreted candidate universe to one bounded exploratory candidate and then to translational association testing. The confirmatory screen remained null, whereas a separately defined exploratory analysis identified one pair meeting the bilateral-SuSiE criteria at the prespecified prior: higher genetically predicted hepatic *LEAP2* expression with higher heel eBMD. The MR association survived a global Bonferroni sensitivity analysis, but the colocalization posterior was prior-sensitive and failed the deliberately conservative 46-analysis stress test. Three liver cell-resolved datasets converged on hepatocytes as the dominant source, and spatial data confirmed tissue-level detection. Two bulk cohorts showed higher hepatic *LEAP2* in fatty liver. Serum LEAP2 was lower in OP than NOP in the age-imbalanced exploratory cohort. In mice, Orx and HFD were accompanied by lower hepatic *Leap2* mRNA and circulating LEAP2 and adverse trabecular phenotypes; robust interaction evidence was confined to TV and Tb.Sp. Pooled liver–serum–bone correlations were largely group-driven. Together, the results support cross-layer association while leaving circulating mediation, independence from confounding, and direct skeletal action unresolved.

### 4.2 What the genetic evidence does—and does not—show

At the prespecified shared-variant prior, the high edge-A posterior was more informative than a single lead-SNP MR result because it compared conditionally resolved signals across a complete regional variant set. Its direction was anchored by harmonized allele effects, not by PP.H4 itself: the rs12520928 A allele increased both hepatic *LEAP2* expression and heel eBMD. The association component was highly stable to conventional multiplicity control, remaining significant after Bonferroni correction across all 1,414 possible signal–outcome combinations (adjusted *p* = 1.39 × 10^−14^).

The distinction between association and colocalization evidence is important. For the selected credible-set pair, PP.H4 declined from 0.9153 at *p*_12_ = 1 × 10^−5^ to 0.8439 at 5 × 10^−6^ and 0.5195 at 1 × 10^−6^; under the deliberately conservative *p*_12_ = 1 × 10^−5^/46 stress test, it fell to 0.1903. The 46-fold prior reduction is not a formally calibrated Bayesian family-wise-error correction, but it demonstrates that the exploratory colocalization classification is prior-sensitive and not experiment-wide multiplicity-robust. Edge B was analytically indeterminate because the circulating-pQTL SuSiE model did not converge; it therefore cannot establish either colocalization or noncolocalization between the liver eQTL and circulating LEAP2. The contrast between a high ordinary-ABF H4 and a nonconverged multisignal fit highlights sensitivity to multisignal modeling and LD specification; it does not provide decisive negative biological evidence. Edge C was compatible with moderate sharing, but the evidence was below the defined threshold for strong sharing. Even if all three pairwise edges had passed, that pattern would represent genetic triangulation rather than formal mediation. In the present data, LEAP2 remains a hypothesis-generating candidate, and a liver-expression–circulating-protein–bone pathway remains unestablished.

### 4.3 Hepatocyte origin and metabolic-liver context

The hepatocyte-source result was reproducible across different cell-resolved technologies and analysis structures and is consistent with the original description of LEAP2 as a liver-expressed peptide and with newer metabolic studies (15, 18, 22). Importantly, source metrics were not treated as interchangeable: GSE174748 and GSE330266 quantified hepatocyte shares of raw *LEAP2* counts, whereas GSE192740 quantified the hepatocyte share of *LEAP2*-positive whole-liver nuclei. Agreement supports the restrained term hepatocyte-enriched, not liver-specific.

The directionally discordant human and mouse findings require more specific interpretation than a generic statement of context dependence. In both bulk-liver cohorts, hepatic *LEAP2* was higher in established NAFL/NASH, and GSE126848 showed a graded increase from histologically normal obesity to NAFL and NASH. An independent integrative analysis similarly reported *LEAP2* upregulation in human MASH hepatocytes (22). These observations are compatible with a chronic hepatocyte response to sustained overnutrition and altered insulin-glucagon signaling, but they do not demonstrate that hepatic lipid accumulation itself induces *LEAP2*. The human biopsies represented clinically heterogeneous disease of uncertain duration and may differ in obesity, insulin resistance, medication exposure, inflammation, fibrosis, and cellular composition. These factors are plausible sources of heterogeneity rather than demonstrated mediators in our data; whole-liver transcript abundance may integrate disease stage and tissue remodeling rather than a single metabolic stimulus.

By contrast, the mouse experiment used young males subjected to a synchronized 12-week HFD exposure and abrupt androgen deprivation. Factorial analysis demonstrated independent Orx effects on hepatic *Leap2* expression and circulating LEAP2; consequently, the reductions in hepatic *Leap2* mRNA and circulating LEAP2 in mice cannot be attributed to HFD or steatosis alone. Direct androgenic regulation of LEAP2 has not been established, so Orx is considered a model-specific endocrine modifier rather than a demonstrated mechanism. Nutritional state provides an additional but incomplete explanation. Published experiments show that plasma LEAP2 decreases during fasting and rises after refeeding, while meal composition can alter hepatic or intestinal *Leap2* expression and circulating peptide; insulin-receptor antagonism attenuates the postprandial response in mice, and glucagon infusion lowers circulating LEAP2 in humans (22, 48, 49). Nevertheless, all four mouse groups underwent the same 12-h fast, so fasting may affect cross-study comparability and absolute serum concentrations but cannot by itself explain the HFD and Orx contrasts. Moreover, a 24-h fast reduced mouse plasma LEAP2 without changing hepatic *Leap2* mRNA, whereas diet-induced obesity increased plasma LEAP2 despite unchanged hepatic expression (48). Human bulk-liver RNA, mouse hepatic qPCR, and mouse serum ELISA are therefore not interchangeable measures; secretion and processing, intestinal contribution, distribution, and clearance may uncouple circulating peptide from hepatic transcription. The directional discrepancy remains unresolved and requires standardized fed-fasted time-course studies with paired hepatic RNA, hepatic protein, and circulating LEAP2 measurements, ideally combined with androgen replacement or LEAP2-targeted intervention.

The negative or nonreplicated pathway correlations are also informative. A perfect four-donor inverse fatty-acid correlation in GSE192740 (*ρ* = − 1.00, exact *p* = 0.0833) and a GSE126848-specific insulin-response association are too sample- or cohort-dependent to support a causal metabolic pathway. They are appropriately retained as supplementary context rather than used to decorate the primary LEAP2 narrative.

### 4.4 Mechanistic boundary at the bone side

LEAP2 is known as a GHSR ligand, but ligand–receptor knowledge alone does not prove that bone cells are direct targets. Here, two human bone single-cell datasets contained no raw GHSR counts, and the final osteoblast bulk dataset could not establish expression above platform background. These data cannot exclude extremely rare cells, transient states, or expression below the sensitivity of the assays. They do, however, preclude a confident direct LEAP2–GHSR osteoblast or osteoclast mechanism from the available public transcriptomes. A systemic metabolic, neuroendocrine, or indirect liver–bone pathway remains possible but is not demonstrated by the current analyses.

### 4.5 Interpretation of the serum and animal findings

The clinical finding was directionally consistent with the genetic expectation, but its magnitude requires conservative interpretation. OP participants were substantially older, BMI and metabolic covariates were unavailable, age-appropriate DXA readjudication was incomplete, and the full-cohort LEAP2–T-score correlation disappeared within each group. Firth regression reduces small-sample separation bias (40, 41) but cannot remove residual confounding or limited covariate overlap. Accordingly, although the association persisted in an age- and sex-adjusted exploratory model, residual confounding precludes an independent association claim. In mice, lower hepatic *Leap2* mRNA and circulating LEAP2 accompanied adverse trabecular phenotypes, but only TV and Tb.Sp showed supported HFD-by-Orx interactions, and pooled correlations attenuated after accounting for group structure. The experiments establish association across conditions, not causation or rescue.

### 4.6 Strengths and limitations

Major strengths include a defined candidate universe, fixed FDR denominators, explicit separation of confirmatory and exploratory layers, complete regional variant analysis, bilateral fine-mapping, predefined analytical failure rules, and donor-level public-data inference. The study also reports unresolved and disconfirming evidence directly: the pQTL triangle was incomplete, edge B was analytically indeterminate because of nonconvergence, spatial zonation was indeterminate, and public bone transcriptomes did not establish GHSR expression. Technical-well measurements were reduced to participant- or animal-level means, the mouse design was analyzed factorially, and pooled animal correlations were audited against within-group and covariate-adjusted models.

Several limitations are material. *LEAP2* was supported primarily by heel eBMD, an ultrasound-derived estimate, rather than replicated primary DXA outcomes and therefore remains an exploratory E-S2 candidate. Although the *LEAP2*–heel eBMD MR association survived Bonferroni correction across 1,414 possible signal–outcome tests, the colocalization posterior was prior-sensitive and did not meet the exploratory threshold under the deliberately conservative 46-analysis prior stress test. The heterogeneous E1–E3 entry rules were not calibrated to a common experiment-wise error rate, and the downstream Bayesian analyses lacked a prespecified hierarchical multiplicity model; *LEAP2* therefore cannot be considered an experiment-wide confirmatory finding. The liver eQTL–circulating pQTL edge was unresolved because the pQTL model did not converge, and the LD reference may not fully represent all contributing populations. The human serum analysis was a small hospital-based convenience sample with marked age imbalance, limited covariate overlap, no independent external validation, and unavailable BMI and metabolic covariates. NOP included participants with osteopenia. Accordingly, the clinical result is descriptive and cannot establish diagnostic performance or an independent association.

The young-male HFD/Orx factorial stress model is not a clinical analogue of age-related or postmenopausal osteoporosis. It combined metabolic exposure with abrupt surgical androgen deficiency and therefore cannot explain the findings from the older, predominantly female clinical comparison. The animal experiment did not include LEAP2-targeted or hepatocyte-specific gain- or loss-of-function, recombinant-protein administration, or rescue. Cell-based experiments were also not performed. Consequently, the present findings cannot establish that liver-derived LEAP2 mediates the skeletal phenotype; cellular targets, receptor dependence, downstream signaling, and direct effects on osteoblasts, osteoclasts, or their precursors remain unresolved.

### 4.7 Implications and future directions

The next decisive step is prospective perturbation of LEAP2 *in vivo*. Liver-targeted overexpression or suppression, or controlled administration of recombinant LEAP2, should establish whether changes in hepatic and circulating LEAP2 precede and modify skeletal outcomes. Such studies should include prespecified micro-CT and histomorphometric endpoints, metabolic and androgen-status stratification, and direct measurement of circulating peptide. Given that the current public bone transcriptomes did not support a direct bone-cell GHSR mechanism, future work should also distinguish direct skeletal signaling from systemic, neuroendocrine, and metabolic pathways. Independent clinical cohorts with age matching, BMI and metabolic covariates, site-specific BMD, and prospective fracture outcomes will be required before LEAP2 can be evaluated as a biomarker or therapeutic target.

## 5 Conclusion

The confirmatory liver-secreted-gene screen was null. In a separately defined exploratory layer, *LEAP2*–heel eBMD was the sole pair meeting the fixed SuSiE criteria at the prespecified prior; the MR association survived a *post hoc* global Bonferroni sensitivity analysis, but the colocalization posterior did not meet the exploratory threshold under a deliberately conservative 46-analysis prior stress test. Human liver datasets established hepatocyte-enriched expression, serum LEAP2 was lower in the exploratory OP cohort, and HFD/Orx-associated trabecular deterioration in mice was accompanied by lower hepatic *Leap2* mRNA and circulating LEAP2. These findings nominate a prior-sensitive, hypothesis-generating candidate but do not establish a complete liver-expression–circulating-protein–bone pathway, direct bone-cell GHSR signaling, diagnostic utility, or causal bone protection by LEAP2.

## Data Availability

The datasets presented in this study can be found in online repositories. The names of the repository/repositories and accession number(s) can be found in the article/**Supplementary Material**.
