# Supplementary material for: Hepatocyte-enriched LEAP2 in relation to heel bone density and osteoporosis-related phenotypes: an exploratory genetic and translational study
Source: Front Endocrinol (Lausanne). 2026 Oct 30;17:1959499. doi: 10.3389/fendo.2026.1959499 (PMC13759347; doi:10.3389/fendo.2026.1959499)
Supplement: Supplementary file 1 [file Supplementaryfile1.docx]

**Supplementary Material**

*Hepatocyte-enriched LEAP2 and osteoporosis-related phenotypes*

# Supplementary Table S1. Prespecified skeletal GWAS outcomes

| **Trait** | **Role** | **Source / identifier** | **Phenotype** | **Ancestry** | **N (cases/controls)** | **Build / citation** |
| --- | --- | --- | --- | --- | --- | --- |
| Osteoporosis | Primary | FinnGen R11 M13_OSTEOPOROSIS | Clinical osteoporosis | Finnish-European | 438,872 (9,046/429,826) | GRCh38; ref 34 |
| Fragility fracture | Primary | FinnGen R11 OSTEOPOROSIS_FRACTURE_FG | Osteoporosis-related fracture | Finnish-European | 343,460 (2,085/341,375) | GRCh38; ref 34 |
| Femoral-neck BMD | Primary | GEFOS2 FNBMD POOLED GC | Femoral-neck BMD | European | 32,961 | GRCh37; ref 31 |
| Lumbar-spine BMD | Primary | GEFOS2 LSBMD POOLED GC | Lumbar-spine BMD | European | 32,961 | GRCh37; ref 31 |
| Total-body BMD | Primary | GCST005348 | Total-body BMD | Predominantly European | 66,628 | GRCh37; ref 32 |
| Heel eBMD | Supportive | GCST006979 | Ultrasound-derived heel eBMD | European UK Biobank | 426,824 | GRCh37; ref 33 |
| General fracture | Supportive | GCST006980 | Incident / prevalent fracture | European UK Biobank | 426,795 (53,184/373,611) | GRCh37; ref 33 |

Effect sizes for femoral-neck and lumbar-spine BMD were reconstructed from signed statistics because original beta and standard error were unavailable; this was flagged in the audit trail. Exact source-file hashes are retained with the analysis manifest.

# Supplementary Table S2. Interaction-focused mouse simple effects

| **Outcome** | **Predefined contrast** | **Estimate** | **95% CI** | **Holm P** |
| --- | --- | --- | --- | --- |
| TV (mm3) | Orx vs Sham under chow | +0.118 | -0.233 to 0.468 | 0.496 |
| TV (mm3) | Orx vs Sham under HFD | +1.581 | 1.274 to 1.888 | 8.91 x 10^-11 |
| TV (mm3) | HFD vs chow under Sham | +0.500 | 0.226 to 0.774 | 0.00171 |
| TV (mm3) | HFD vs chow under Orx | +1.963 | 1.586 to 2.340 | 8.91 x 10^-11 |
| Tb.Sp (mm) | Orx vs Sham under chow | +0.0376 | 0.0191 to 0.0561 | 0.000269 |
| Tb.Sp (mm) | Orx vs Sham under HFD | +0.1281 | 0.1040 to 0.1522 | 4.29 x 10^-11 |
| Tb.Sp (mm) | HFD vs chow under Sham | +0.0777 | 0.0596 to 0.0957 | 2.82 x 10^-9 |
| Tb.Sp (mm) | HFD vs chow under Orx | +0.1681 | 0.1436 to 0.1925 | 1.26 x 10^-13 |

# Figure S1. Evaluation of GHSR expression in public human bone transcriptomes.

The audited bone single-cell resources contained GHSR in the feature annotation but zero raw counts across 51,489 filtered cells from six donors. In GSE156508, the mapped GHSR transcript cluster overlapped the low-intensity background range and the osteoporotic-fracture versus severe-osteoarthritis contrast was null (mean difference -0.068; 95% CI -0.345 to 0.209; Welch P=0.586). These data do not convincingly support direct bone-cell GHSR expression.

# Figure S2. Age- and sex-matched clinical sensitivity analysis.

Seven exact-sex nearest-age pairs within a 5-year caliper are connected by lines. The paired LEAP2 difference remained directionally consistent (paired Wilcoxon P=0.031), but the small matched sample demonstrates limited covariate overlap and cannot resolve confounding.

# Figure S3. Exploratory individual-animal liver-serum-bone correlations.

Hepatic Leap2 relative expression versus serum LEAP2, and serum LEAP2 versus BV/TV and Tb.Sp. Overall Spearman estimates are shown. Within-group and factorial-adjusted analyses were not consistently supportive, indicating that pooled correlations largely reflect between-group separation.
